# Evaluation of Plant Growth-Promoting and Salinity Ameliorating Potential of Halophilic Bacteria Isolated From Saline Soil

**DOI:** 10.3389/fpls.2022.946217

**Published:** 2022-07-15

**Authors:** Chintan Kapadia, Nafisa Patel, Ankita Rana, Harihar Vaidya, Saleh Alfarraj, Mohammad Javed Ansari, Abdul Gafur, Peter Poczai, R. Z. Sayyed

**Affiliations:** ^1^Department of Plant Molecular Biology and Biotechnology, ASPEE College of Horticulture and Forestry, Navsari Agricultural University, Navsari, India; ^2^Naran Lala College of Professional and Applied Sciences, Navsari, India; ^3^Zoology Department, College of Science, King Saud University, Riyadh, Saudi Arabia; ^4^Department of Botany, Hindu College (Mahatma Jyotiba Phule Rohilkhand University Bareilly), Moradabad, India; ^5^Sinarmas Forestry Corporate Research and Development, Perawang, Indonesia; ^6^Finnish Museum of Natural History, University of Helsinki, Helsinki, Finland; ^7^Department of Entomology, Asian PGPR Society for Sustainable Agriculture, Auburn University, Auburn, AL, United States; ^8^Department of Microbiology, PSGVP Mandal's‘S I Patil Arts, G B Patel Science, and STKV Sangh Commerce College, Shahada, India

**Keywords:** abiotic stress, ACC deaminase, antioxidant enzymes, biofilm, IAA, microbial diversity, PGPR, saline soil

## Abstract

Among the biotic and abiotic stress affecting the physical, chemical, and biological properties of soil, salinity is a major threat that leads to the desertification of cultivable land throughout the world. The existence of diverse and versatile microbial populations inhabiting the nutrient-rich soil and varied soil conditions affects the soil dynamism. A normal soil constitutes 600 million bacteria belonging to about 20,000 species, which is reduced to 1 million with 5,000–8,000 species in stress conditions. Plant growth-promoting rhizobacteria (PGPR) are in symbiotic association with the plant system, which helps in combating the abiotic stress and increases the overall productivity and yield. These microorganisms are actively associated with varied cellular communication processes through quorum sensing and secondary metabolites such as the production of Indole-3-acetic acid (IAA), exopolysaccharide (EPS) siderophore, ammonia, ACC deaminase, and solubilization of phosphate. The present study focused on the isolation, identification, and characterization of the microorganisms isolated from the seacoast of Dandi, Navsari. Twelve isolates exhibited PGP traits at a high salt concentration of 15–20%. AD9 isolate identified as *Bacillus halotolerans* showed a higher ammonia production (88 ± 1.73 μg/mL) and phosphate solubilization (86 ± 3.06 μg/mL) at 15% salt concentration, while AD32^*^ (*Bacillus* sp. clone ADCNO) gave 42.67 ±1.20 μg/mL IAA production at 20% salt concentration. AD2 (*Streptomyces* sp. clone ADCNB) and AD26 (*Achromobacter* sp. clone ADCNI) showed ACC deaminase activity of 0.61 ± 0.12 and 0.60 ± 0.04 nM α-ketobutyrate/mg protein/h, respectively. AD32 (*Bacillus* sp. clone ADCNL) gave a high siderophore activity of 65.40 ± 1.65%. These isolates produced salinity ameliorating traits, total antioxidant activities, and antioxidant enzymes *viz*. superoxide dismutase (SOD), Glutathione oxidase (GSH), and catalase (CAT). Inoculation of the multipotent isolate that produced PGP traits and salinity ameliorating metabolites promoted the plant growth and development in rice under salinity stress conditions. These results in 50% more root length, 25.00% more plant dry weight, and 41% more tillers compared to its control.

## Introduction

Agriculture has been adversely affected by abiotic stress such as salinity, drought, temperature, and so on. These are major hindrances in sustainable agriculture all around the globe (Gupta and Pandey, 2019; Kannepalli et al., 2020; Kapadia et al., 2021). Light, temperature, drought, salinity, soil, air, and water pollutants are the major abiotic stress conditions of major concern. High temperature during the night is concluded to cause a significant reduction in metabolites and plant growth hormones. Temperature stress also alters antioxidant activities and the production of ROS affecting the rice cultivars (Al-Zahrani et al., 2022).

Soil salinization is a serious threat to productivity, food security, and economic development by causing a reduction in the net cultivable area (Sagar et al., 2020, 2022a,b; Nasab et al., 2022). Arid and Semi-arid regions are more prone to salinization due to higher evaporation rates and lesser fresh water to remove the salts (Fazeli and Sayyed, 2019). High salinity has affected around 20% of the total cultivated areas and 33% of irrigated agricultural land by 2050. The estimated arable areas to be salinized account for more than 50% (Shrivastava and Kumar, 2015; Singh, 2022). About 23 million acres of land are affected by salinity/alkalinity/acidification worldwide. In India, the area under salt-affected soil spreads over an estimate of 6.73 Mha, covering many states (Sharma et al., 2015, 2016; Gopalakrishnan and Kumar, 2020).

Soils that have excessive amounts of salts (i.e., electrical conductivity (EC) > 4 dS/m) are classified as saline soils (approximately 40 mM NaCl) at 25°C and have exchangeable sodium of 15% (Thomas and Pierzynski, 2018). A drastic increase in salinized areas is witnessed for various reasons, including low precipitation, high surface evaporation, weathering of native rocks, irrigation with saline water, and poor cultural practices. Salinization affects productivity, making it challenging to meet the food demands against the increasing world population. Salinity hampers the development of plants at all stages, including germination, vegetative growth, and reproductive development. It enhances programmed cell death in some tissue types, ovule abortion, and senescence of fertilized embryos (Kamran et al., 2020). Soil salinity imposes ion toxicity, low osmotic potential and nutritional (N, Ca, K, P, Fe, Zn) deficiency (Su et al., 2021), oxidative stress on plant development by either reduction or cessation in the metabolic processes and photosynthesis, respiration, nitrogen fixation (Kasim et al., 2016), and reduces water availability to plants (Safdar et al., 2019). Salinity also affects photosynthesis mainly through a reduction in leaf area, chlorophyll content, stomatal conductance, and to a lesser extent, a decrease in photosystem II efficiency (Saddiq et al., 2021). Salt stress conditions lead to serious implications on the plant growth resulting in huge loss, hence many mitigation strategies are formulated for sustainable agriculture. Plant growth-promoting microorganisms are natural tools to combat salinity stress conditions (Sabagh et al., 2020). Much work is also focused on the use of arbuscular mycorrhizal fungi and its symbiotic association with plants to increase plant growth. In one such study, AMF was used along with zinc for growth of maize plant as well as its role to overcome Zn toxicity and growth enhancement in maize plants (Saboor et al., 2021).

Plant growth-promoting rhizobacteria (PGPR) are known to promote plant growth through various mechanisms (Kalam et al., 2020; Kannepalli et al., 2020; Basu et al., 2021; Hamid et al., 2021; Khan et al., 2021; Manasa et al., 2021). PGPR is one of the best alternatives to alleviate the salinity effect on plants. These microbes of various genera have been reported to enhance plant survival and its growth in the saline environment by means of producing biofilms (Nasab et al., 2022), indole-3-acetic acids (IAA) (Khairnar et al., 2022), exopolysaccharide (EPS) (Sayyed et al., 2015; Ilyas et al., 2020), aminocyclopropane-1-carboxylate (ACC) deaminase (Ilyas et al., 2020; Sagar et al., 2020), siderophore (Sayyed et al., 2005, 2019; Wani et al., 2016; Patel et al., 2018; Nithyapriya et al., 2021), ammonia, and through P solubilization (Taylor et al., 2007; Sharma et al., 2013). Salt tolerant PGPR are studied for applying them to increase the productivity of the salt-affected land. These organisms can be used as bioinoculants instead of chemical-based neutralizers because of their salt combating capabilities such as producing compatible solutes and transporters. The present study is focused on the isolation of PGPR from the saline coast of Dandi, India, and characterized for their plant growth-promoting traits to understand the role of these isolates in plant growth and development. PGPR plays a substantial role in the vegetative growth, nutrient contents, increased root growth, and stem length.

## Materials and Methods

### Collection of Soil Sample

Rhizosphere soil samples were collected in sterile containers with sterile rods and forceps from the Arabian sea coasts, Dandi, Navsari, Gujarat, India (Latitude: 20.89 North, Longitude: 72.81 East) at a depth of 1 to 1.6 ft. These samples were transported to the laboratory and stored at 4°C until further use (Passari et al., 2017).

### Isolation and Identification of Isolates

#### Morphological and Cultural Identification

The samples were serially diluted up to 10^−5^, and 100 μL of each dilution was plated on the Luria–Bertani (LB) agar medium (Hi media) as well as on the Potato Dextrose Agar (PDA; Himedia) and incubated at 30°C for 24–48 h. The well-isolated and pure colonies were analyzed for their morphological and biochemical traits according to Bergey's Manual of Determinative Bacteriology (Norman et al., 1974). Upon incubation, bacterial colonies with unique cultural characteristics were further sub-cultured and stored at glycerol stock for further use.

#### Molecular Identification of Isolates

The genomic DNA of each isolate was extracted by the LPS buffer method (Nilpa et al., 2022) and the quality of isolated DNA was checked on 1% agarose gel prepared in 1X TAE buffer. The universal PCR primers 27F (52 AGA GTT TGA TCC TGG CTC AG 3G) and 1492R (52 TAC CTT GTT ACG ACTT 3AC) were used to amplify the highly conserved ~1,400 bp region of the 16S ribosomal RNA gene (rRNA gene). Primers ITS1 (5'-TCCGTAGGTGAACCTGCGG-3') and ITS4 (5' TCCTCCGCTTA TTGATATGC 3') were used to amplify the ~648 bp region for fungal isolates. Each 50 μl of PCR reaction mixture contained 20 ng of genomic DNA, 200 μM of each deoxynucleotide triphosphate (dNTP), 5 μL of buffer, 1.5 mM MgCl_2_, 10 picomoles of each primer, and 1 unit of Taq polymerase (SLS Research Ltd., Surat, India). Amplification was confirmed by the presence of an intense single band of respective sizes. The amplified PCR product was purified using a DNA purification kit (SLS Research Ltd., Surat, India) and subjected to the ABI 3130xl genetic analyzer (Applied Biosystems, CA) for sequencing. The obtained gene sequence was compared with GenBank submissions using the BLASTn program and sequences were submitted to the NCBI using BankIt tool to assign accession numbers (https://www.ncbi.nlm.nih.gov). The phylogenetic analysis was done by the Molecular Evolutionary Genetics Analysis (MEGA-7) software (www.megasoftware.net).

### Screening of Isolates for Their Plant Growth-Promoting Traits

#### Ammonia Production

The 50 μL overnight grown cultures (OD^600^ 0.2) were inoculated in 1% peptone broth (HiMedia) and incubated at 37°C in a shaking incubator at 150 rpm for 48 h. A 1-mL Nessler's reagent was added to each tube, the development of a brown to the yellow color indicated a positive test, while the intensity of the color indicated the amount of ammonia produced by the isolates (Cappucino, 1987).

#### Screening and Estimation of Phosphate Solubilization

For qualitative estimation of P solubilization, the isolated pure microbes were tested for their ability to solubilize phosphate on Pikovaskay's medium supplemented with pH indicator (Bromophenol Blue; 0.025 mg/ml) (Mehta and Nautiyal, 2001) and 1 g/L tricalcium phosphate as a substrate. The overnight grown cultures were plated on the Pikovskaya's medium and incubated at 30°C for 72 h. The clear zone around the colonies indicated a positive response. The isolates showed that large halo zones were further evaluated for their ability to solubilize phosphate quantitatively in liquid media. The liquid Pikovskaya's medium containing tricalcium phosphate was inoculated (OD^600^ 0.2) with selected isolates (AD1, AD2. AD3, AD4, AD8, AD9, AD11, AD26, AD28, AD31, AD32, AD22, AD29, AD32^*^, AD38) and incubated in a shaker incubator at 150 rpm for 10 to 12 days. After every 2 days of incubation, small aliquots have been withdrawn aseptically to know the growth and pH of the media. The soluble phosphate concentration in the supernatant was estimated by the vanado molybdophosphoric yellow color method (Subha et al., 1988).

#### Siderophore Production

The overnight grown culture with uniform densities was inoculated in a succinate medium and incubated at 30°C in a shaker incubator for 24 h. The overnight grown culture was centrifuged, and the clear supernatant was mixed with a 0.5-mL CAS reagent. The OD was measured after 20 min at 630 nm to estimate the siderophore production (Schwyn and Neilands, 1987; Jenifer and Sharmili, 2015).

#### Biofilm Formation

Isolates were grown in LB medium for 48 h at 30°C; after incubation, the cultures with used media were discarded. The crystal violet (0.5%) solution was added to the tube and incubated for 5 min. The solution was discarded and tubes were washed with distilled water. The biofilm was noticed by the adherence to color (Kirmusaoglu, 2019).

### Screening for Salinity Ameliorating Metabolites

#### Production of Aminocyclopropane-1-Carboxylate Deaminase

ACC deaminase activity was assayed by measuring the amount of α-ketobutyrate produced by the cleavage of ACC through ACCD (Honma and Smmomura, 1978). The number of mM of α-ketobutyrate produced by this reaction is determined by comparing the absorbance at 540 nm of a sample to a standard curve of α-ketobutyrate (0.1 mM-1.0 mM). For ACC deaminase activity assay, isolates were pelleted and washed twice with 5 mL of 0.1 M Tris-HCl (pH 7.5). About 6 mL of minimal salt media along with 600 μL of 0.1 M Tris-HCl (pH 8.5) was added, followed by the addition of 30 μL of 0.5% toluene and vortexed for 30 s. The 200 μL of these cells were taken in experimental and control tubes and 10 μL of ACC (0.3 mM) was added only in experimental tube. About 0.1 M Tris-HCl (pH 8.5) and 13 μL of ACC (0.3 mM) were added to each tube along with a blank. The tubes were kept in the water bath at 34Â°C for 30 min, after which 0.56 N HCl was added to each tube and vortexed for 30 s, centrifuged for 12 min at 1,200 rpm, and the supernatant was transferred to their respective tubes, followed by the addition of 800 μL of 0.56 N HCl. The 300 μL of 2, 4-dinitrophenyl hydrazine (DNF) was added to each tube except blank, vortexed, and kept in a water bath for 30 min at 30°C. A 2-mL of NaOH was added, followed by a measurement of OD at 540 nm.

#### IAA Production

Overnight grown cultures of the isolates were inoculated in LB broth containing 0.1% of tryptophan and incubated at 30°C for 3 days, and 2 mL of the culture were centrifuged at 10,000 rpm for 10 min. The supernatant was taken and mixed with 4 mL of Salkowski's reagent, followed by incubation of 10 min (Patten and Glick, 2002). The intensity of color was measured on a spectrophotometer (Shimadzu 2600) at 535 nm, and the IAA production of each organism was calculated from a standard IAA curve (10-100 μg/ml) (Gordon and Weber, 1951).

#### Screening for Antioxidant Activity of Isolates

Total antioxidant capacity was measured colorimetrically by the phosphomolybdenum method (Prieto et al., 1999). An aliquot of 0.1 mL reagent (0.6 M sulphuric acid, 28 mM sodium phosphate, and 4 mM ammonium molybdate) was dispensed in a tube and incubated at 95°C for 90 min. The sample was allowed to cool down to room temperature, and then the absorbance was measured at 695 nm. A typical blank solution containing 1.0 mL of reagent solution and the appropriate volume of the same solvent used for the sample was incubated under the same conditions. Ascorbic acid was used as control, and the TAC was expressed as μg of ascorbic acid equivalent per mL of extract (μg AAE/mL). The TAC was calculated using the following linear equation based on the calibration curve for ascorbic acid.


Y=0.0011X + 0.0121 ((R2 = 0.9963)

where,

Y is the absorbance at 695 nm.

X is concentration of ascorbic acid (μg/mL).

#### Screening for Antioxidant Enzymes

For screening of antioxidant enzymes namely superoxide dismutase (SOD), catalase (CAT), and reduced glutathione oxidase (GSH), each isolate was separately grown in each minimal medium (MM) at 30°C for 24 h at 120 rpm. After 24 h incubation, each broth was centrifuged at 1,000 rpm for 10 min to obtain cell homogenate.

For the estimation of the SOD, CAT, and GSH activity, 100 μL cell homogenate was mixed with 100 μL of pyrogallol solution in EDTA buffer (pH 7.0), 100 μL of hydrogen peroxide in phosphate buffer (pH 7.0), and 100 μL of GSH, respectively, and the absorbance was measured at 420 nm (Marklund and Gudrun, 1974) and 240 nm (Beers and Sizer, 1952; Salbitani et al., 2017), respectively.

One unit of SOD was defined as the amount of SOD required for preventing 50% autooxidation of pyrogallol and it was expressed as IU/mg. One unit of CAT is defined as mM of H_2_O_2_ decomposed/min. GSH activity was measured as the reduction in μM of GSH per min.

### Plant Growth Promotion Study–Pot Assay

To select the potent PGPR halophilic isolate, plant growth promotion studies were conducted at the pot level under greenhouse conditions. The experiments were conducted with each isolate and control (uninoculated medium) in triplicates as a complete randomized block design (RBD). The rice seeds were surface sterilized in 0.2% HgCl_2_ for ± 2 min, then rinsed with sterile distilled water three times, and then germinated on clean straw paper previously moistened with salinized distilled water (0.6 % NaCl). Seeds were planted, covered with straw paper, and rolled up using plastic and allowed for seed germination in an incubator at 28Â°C for 3 days. After 3 days, each rice seedling was immersed into separate bacterial suspensions (10^8^ CFU/mL) of each isolate, planted into pots, and incubated under greenhouse conditions. Following 21 days after sowing (DAS), each plant was uprooted and subjected to the measurement of plant height (cm), root length (cm), plant dry weight (mg), and number of tillers. The best PGPR isolate was selected based on the simple scoring and ranking method.

### Statistical Analysis

All the experiments were performed in triplicates, and a mean of triplicate was further analyzed statistically using Statistical Analysis System software. F-Test was done to show significant effects on tested variables. Finally, it was continued with Duncan Multiple Range Test (DMRT) at *p* < 0.05 (Gomez and Gomez, 1984).

## Results

### Isolation of PGPR

In the present study, 25 isolated colonies were obtained from high saline soil on an LB agar plate supplemented with NaCl. Out of 25 isolated colonies, 12 isolates were selected based on the growth response of varied salt concentrations, and isolates AD1, AD2, AD3, AD4, AD8, AD9, AD11, AD26, AD28, AD31, AD32, AD22, AD29, AD32^*^, and AD38 exhibited distinct cultural characteristics as well as exhibited growth at a concentration of 15% NaCl and 20% NaCl. Colonies were selected based on distinct cultural characterization which included the study of size, shape, elevation, surface, consistency and pigment production, and growth response to varying salt concentrations. The entire experiment was conducted to evaluate the ability of microbes to carry out PGPR activities at different salt concentrations.

### Characterization of Isolates

#### Identification of Isolates

A microscopic study distinguished the isolates as bacteria, actinomycetes, and fungi. Differential staining characterized the organisms as gram-positive and gram-negative rod-shaped organisms. The cultural characteristics varied from small to large-sized, round or irregular-shaped colonies with entire margins and either opaque or translucent. Isolate AD2 exhibited branching filamentous morphology and characteristic dried, powdery colonies while the fungal mounting confirmed the isolates AD22 and AD29 belonging to Aspergillus sp.

#### Molecular Identification of Isolates

**Figure 1:**
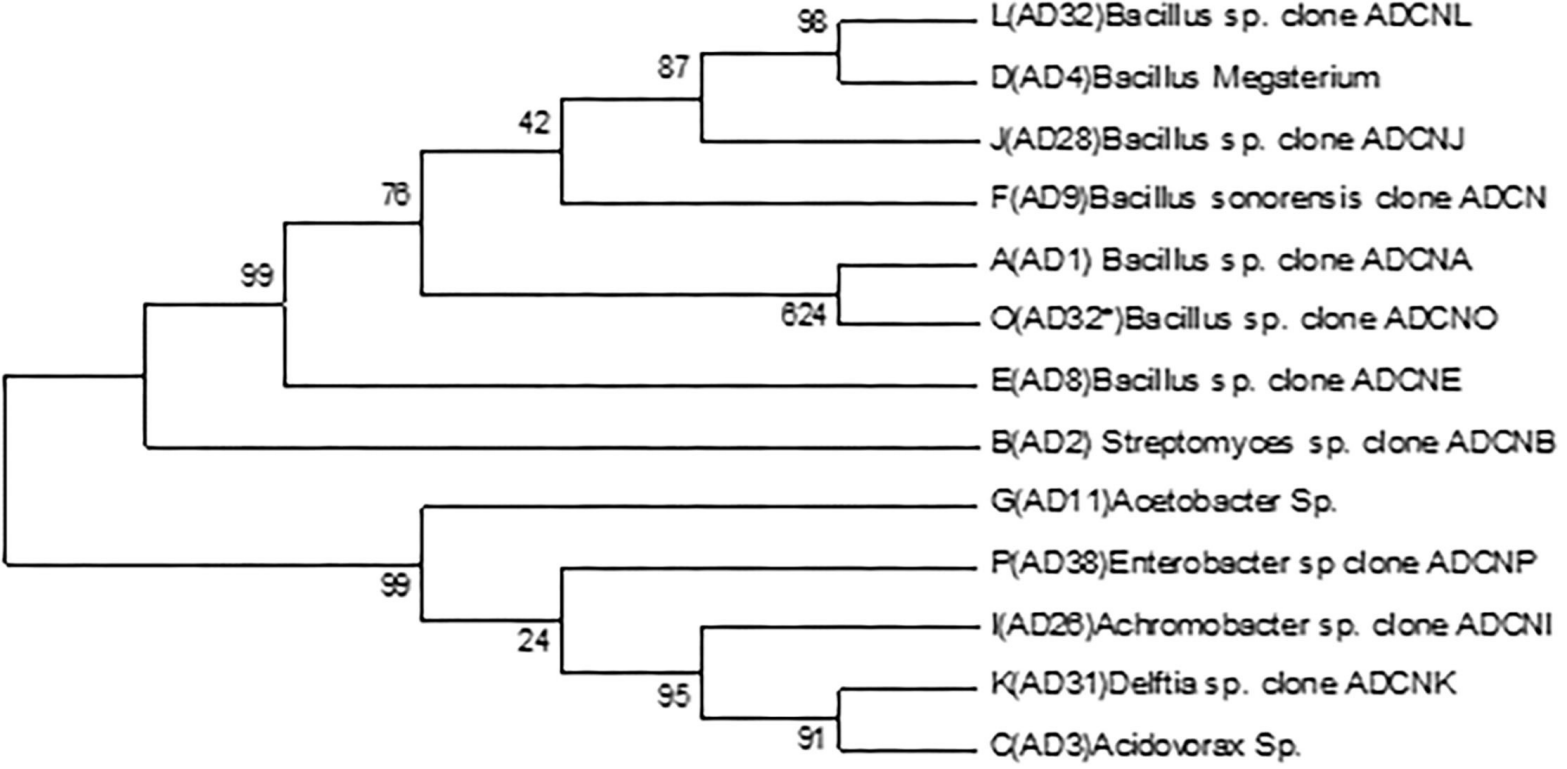
Phylogenetic tree of bacterial isolates based on 16S r RNA gene sequences analyzed based on neighbor-joining method.

**Figure 2:**
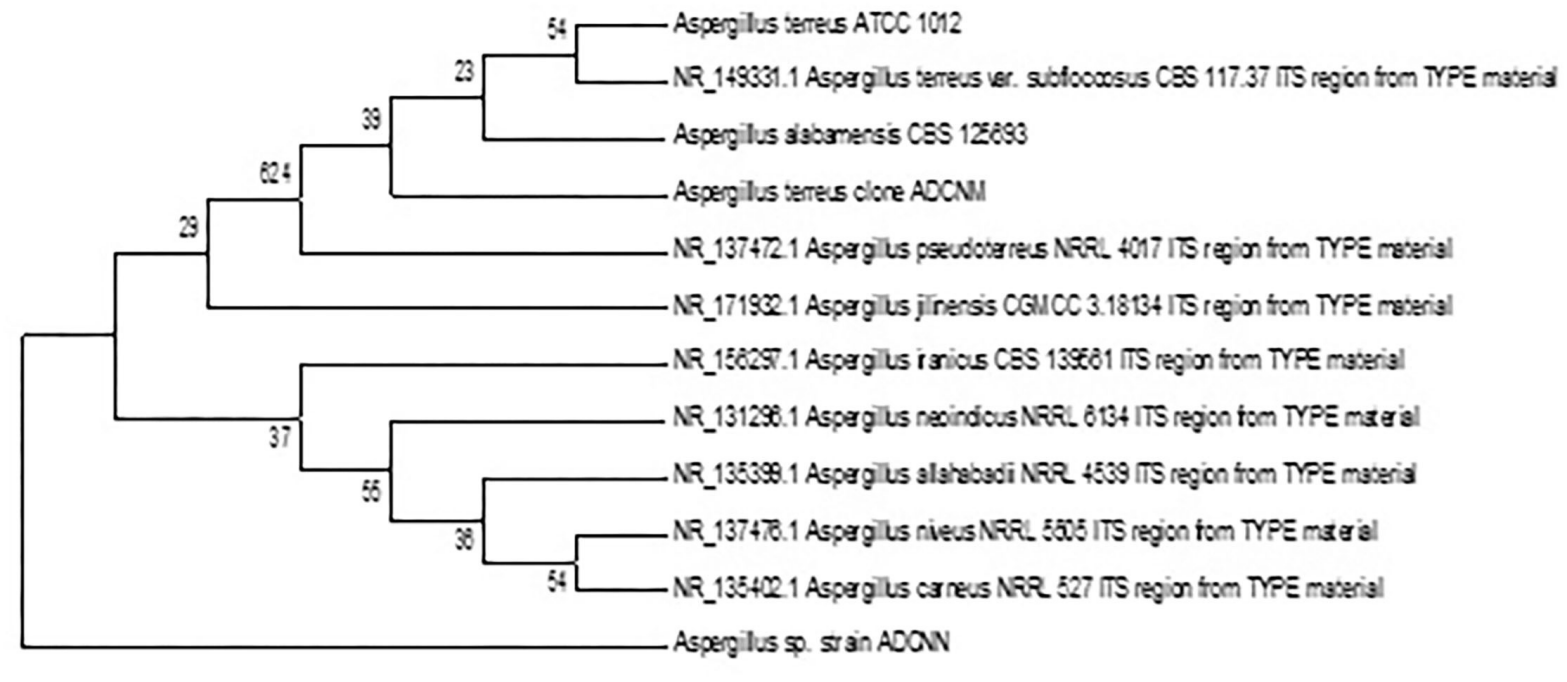
Phylogenetic tree of fungal isolates based on 16S r RNA gene sequences analyzed based on neighbor-joining method.

The accession number was obtained for each isolate identified as *Bacillus* sp. clone ADCNA(AD1), *Bacillus* sp. clone ADCNE (AD8), *Bacillus halotolerans* clone ADCN (AD9), *Bacillus* sp. clone ADCNJ (AD28), *Bacillus* sp. clone ADCNL (AD32), *Bacillus* sp. clone ADCNO (AD32^*^), *Achromobacter* sp. clone ADCNI (AD26), *Delftia* sp. clone ADCNK (AD31), *Enterobacter* sp. clone ADCNP (AD38) (Figure 1), *Streptomyces* sp. clone ADCNB (AD2), *Aspergillus terreus* clone ADCNM (AD22), and *Aspergillus* sp. strain ADCNN (AD29) (Figure 2). The isolates identified were majorly bacteria, actinomycetes, and fungi (Table 1). The phylogenetic tree of the isolates was constructed by using the neighbor-joining method.

**Table 1:** Molecular identification of isolates.

**Code number**	**Accession number**	**Name of the organism**	**Closest organism**	**Identity**	**Accession number**
A (AD1)	MH142384	*Bacillus sp*. clone ADCNA	*Bacillus sonorensis* strain HQB290	98%	KT758474.1
B (AD2)	MH142390	*Streptomyces* sp. clone ADCNB	*Streptomyces enissocaesilis* strain 10B	98%	MF353938.1
E (AD8)	MH142391	*Bacillus* sp. clone ADCNE	*Bacillus cereus* strain SK3	99%	KC857624.1
F (AD9)	MH127536	*Bacillus halotolerans* clone ADCN	*Bacillus sonorensis* strain AH45/ *Bacillus halotolerans* strain FJAT-47799	99%	MG651202.1/ MG651202.1
I (AD26)	MH142385	*Achromobacter* sp. clone ADCNI	*Achromobacter xylosoxidans* strain GYP4	98%	KY697918.1
J (AD28)	MH142386	*Bacillus sp*. clone ADCNJ	*Bacillus mojavensis strain VKAK1*	95%	MF136787.1
K (AD31)	MH142388	*Delftia sp*. clone ADCNK	*Delftia lacustris strain AL100*	98%	MG819361.1
L (AD32)	MH142392	*Bacillus sp*. clone ADCNL	*Bacillus sonorensis* strain BD92	91%	MF767900.1
M (AD22)	MH130061	*Aspergillus terreus* clone ADCNM	*Aspergillus terreus* strain MBL1414	91.67%	KM924436.1
N (AD29)	MH130062	*Aspergillus* sp. strain ADCNN	*Aspergillus sydowii UTHSC* 12-3109	99.03%	LN898733.1
O (AD32^*^)	MH142387	*Bacillus* sp. clone ADCNO	*Bacillus licheniformis* strain CM3HG11	99%	KU664834.1
P (AD38)	MH142389	*Enterobacter* sp. clone ADCNP	*Enterobacter cloacae*	99%	CP020089.1

### Screening for PGP Traits

#### Ammonia Production

**Figure 3:**
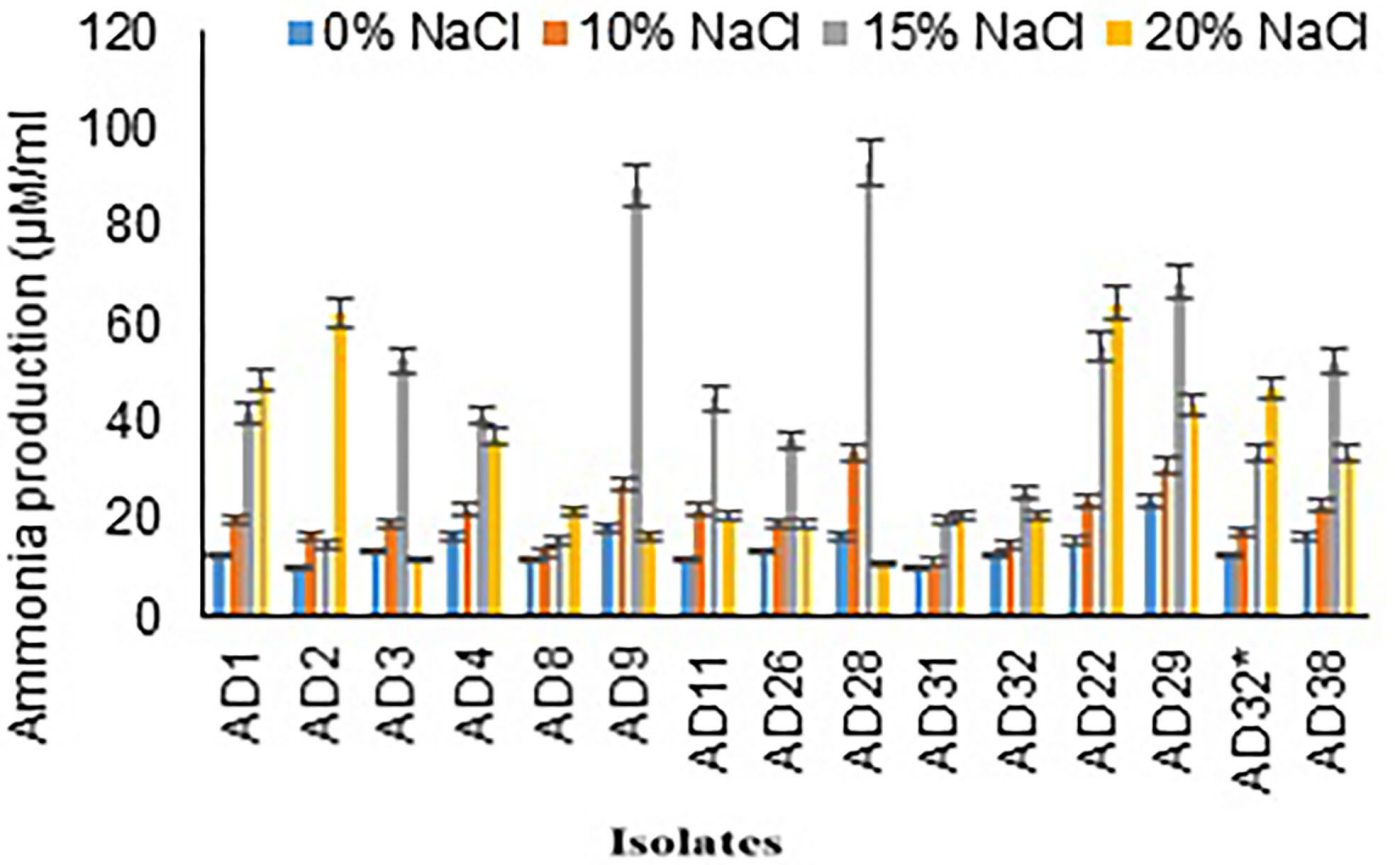
Ammonia production by isolates at different salt concentrations.

The range of ammonia production by selected isolates was 14.67–92.67 μg/mL in 15% NaCl, and it was 10.57–64.00 μg/mL in 20% NaCl. Maximum concentration of ammonia production was observed in isolates AD28 (92.67 μg/mL) and AD9 (88 μg/mL) followed by AD29 (68 μg/mL) and AD22 (55.33 μg/mL) in 15% salt concentration. Few isolates showed a higher ammonia production even in 20% NaCl concentration and exhibited higher production in 20% NaCl concentration as compared to the production of ammonia in 15% salt concentration, which includes AD22 (64 μg/mL), AD2 (62 μg/mL), and AD1 (48.33 μg/mL). Out of 15 isolates, 11 isolates produced more than 30 μg/mL ammonia. Seven isolates exhibited higher ammonia production even in a high salt concentration of 20%, indicative of its adaptation to a halophilic environment. Fungal isolate AD22 64+1.53 μM/mL produced higher ammonia at elevated salt concentrations of 20% compared to bacterial isolates (Figure 3).

#### Phosphate Solubilization

**Figure 4:**
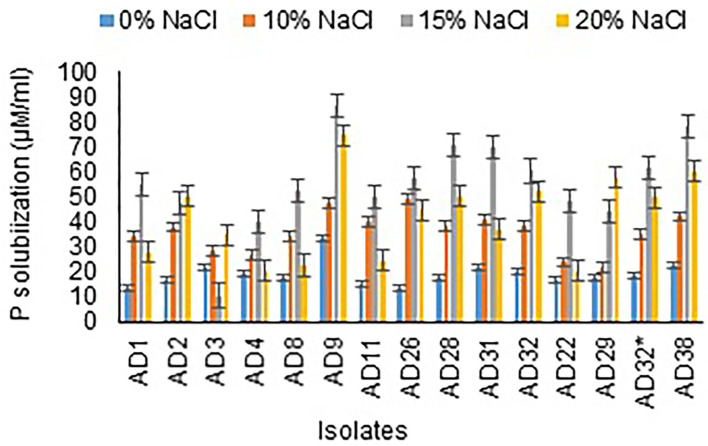
Phosphate solubilization at different salt concentrations.

All the isolates were good phosphate solubilizers and showed a clear zone around the colony after 72 h of incubation. Liquid culture with tricalcium phosphate was used to measure phosphate solubilization by different isolates after 12 days of incubation. The results revealed that AD9 (86 μg/mL), AD38 (78 μg/mL), AD28 (70.33 μg/mL), and AD31 (70 μg/mL) followed by the remaining isolates in 15% NaCl. A noticeable result was observed with AD22, which showed higher solubilization at 20% NaCl concentration than 15% NaCl. The organisms AD9 (74.67 μg/mL) and AD38 (60 μg/mL) gave phosphate solubilization in 20% NaCl, followed by all the other isolates (Figure 4). The salt concentration is observed to have a negative effect on solubilization efficiency, and a similar trend was noticed with phosphate solubilization. All the isolates were better phosphate solubilizers at 15% NaCl concentration.

#### Siderophore Production

Siderophores are iron chelators produced by microbes. In the experimental findings, all the isolates produced siderophores measured by Chrome Azurol Sulphonate (CAS) assay. The siderophore production ranged from 16.03 to 65.4%. The fungal isolates were better producers of siderophores than bacterial isolates except for AD32 (Table 2).

**Table 2:** Production of PGP traits of halophillic isolates.

**Isolates**	**Siderophore production (%)**	**Biofilm formation**
A (AD1)	22.15 ± 1.33	nd
B (AD2)	16.03 ± 0.95	++
E (AD8)	42.20 ± 4.20	nd
F (AD9)	43.20 ± 1.76	++
I (AD26)	32.36 ± 0.78	++
J (AD28)	21.56 ± 2.11	nd
K (AD31)	43.20 ± 0.61	nd
L (AD32)	34.20 ± 1.31	nd
M (AD22)	53.00 ± 2.44	nd
N (AD29)	58.00 ± 1.20	nd
O (AD32^*^)	65.40 ± 1.65	++
(AD38)	24.26 ± 2.13	nd

*Nd, not detected. + = positive, ++ = moderate, +++= strong positive. Values are the average of triplicates and were analyzed by Duncan Multiple Range Test at 5% real level*.

#### Biofilm Formation

Among all the isolates, only a few isolates produce biofilm, viz. AD8, AD9, AD32, and AD38. The biofilm formation is either genetically determined or depends on the culture conditions.

### Salinity Ameliorating Metabolites

#### IAA Production

**Figure 5:**
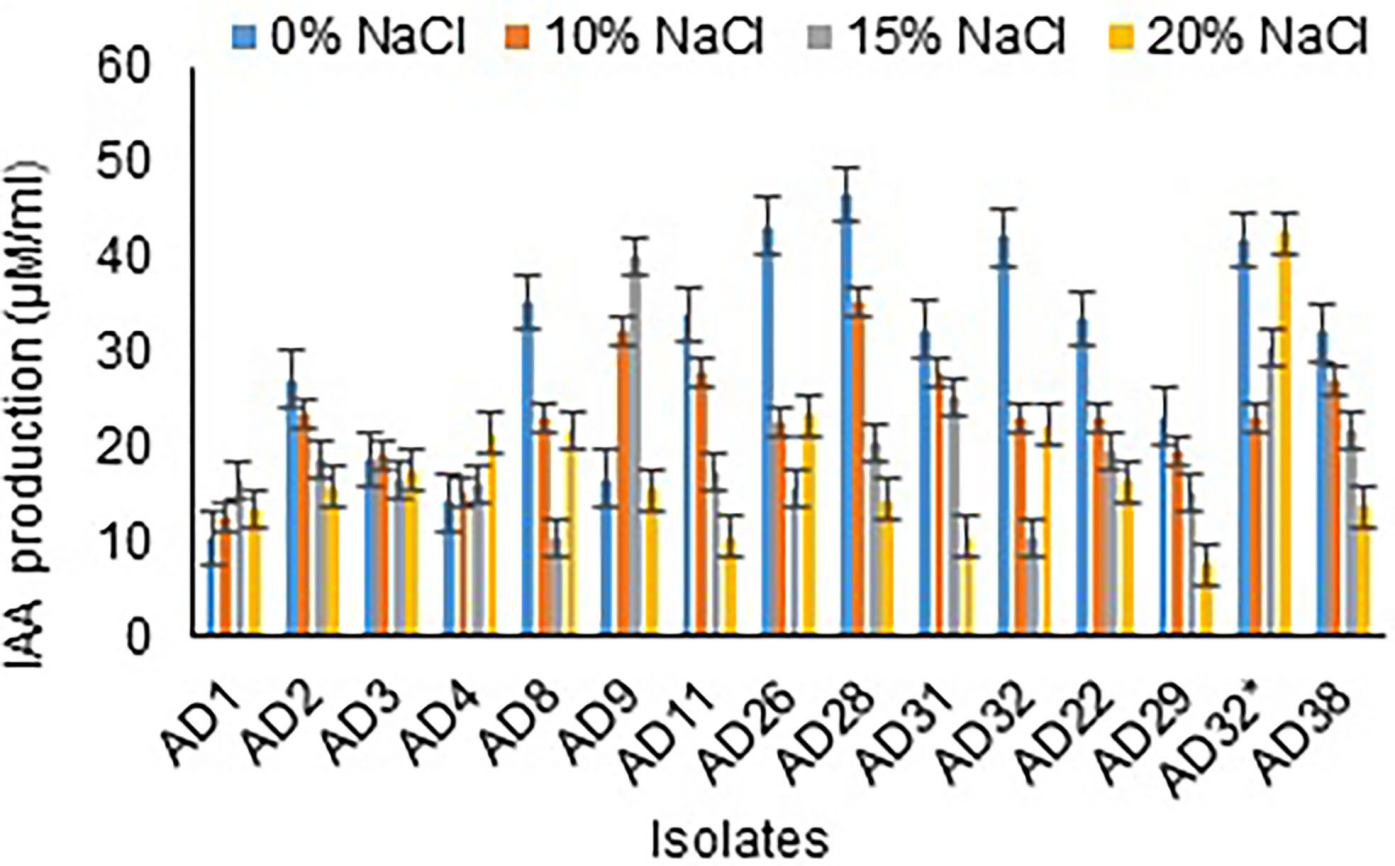
IAA production by isolates at different salt concentrations.

Among 15 isolates, all were observed to produce IAA, ranging from 10.00 to 40.17 μg/mL at 15% NaCl concentration. All the isolates showed either increased or decreased IAA production in 20% NaCl concentration (Figure 5). The isolates AD32^*^ (42.67 μg/mL), AD26 (23.33 μg/mL), and AD8 (21.67 μg/mL) showed IAA production at 15% salt concentration. All the fungal isolates under study either did not produce or comparatively showed less IAA production than the bacterial isolates.

#### ACC Deaminase Activity

ACC deaminase activity enables the plants to withstand adverse environmental conditions and promotes plant growth and development. The highest ACC deaminase activity was exhibited by bacterial strain AD2 (0.61 nmol α-ketobutyrate mg/protein/h) and AD26 (0.60 nmol α-ketobutyrate mg/protein/h), followed by AD31 (0.46 nmol α-ketobutyrate mg/protein/h) (Table 2). ACCD activity occurred at all NaCl concentrations, however, maximum ACCD activity was exhibited by all the isolates at 15% NaCl concentration.

#### Total Antioxidant Capacity

Among the various isolates, four isolates namely AD2, AD9, AD26, and AD32^*^ produced varying amounts of total antioxidant activity. Isolate AD32^*^ produced maximum total antioxidant activity of 14.60±0.18 AAE/mL compared to the other four isolates (Table 3).

**Table 3:** Production of salinity ameliorating traits halophillic isolates.

**Isolates**	**Total antioxidant activity (μg AAE/mL)**	**Antioxidant enzymes**		
		**SOD (IU/mg)**	**CAT (mM of H_2_O_2_ decomposed/min)**	**GSH (μM GSH reduced/min)**
A (AD1)	7.6 ± 0.10	nd	nd	nd
B (AD2)	6.31 ± 0.20	15.19 ± 0.03	0.705 ± 0.02	25.12 ± 0.01
E (AD8)	7.35 ± 0.10	nd	nd	nd
F (AD9)	13.50 ± 0.14	11.01 ± 0.01	0.870 ± 0.01	27.18 ± 0.02
I(AD26)	13.05 ± 0.18	12.18 ± 0.02	0.881 ± 0.03	26.26 ± 0.03
J (AD28)	10.41 ± 0.20	nd	nd	nd
K (AD31)	11.15 ± 0.10	nd	nd	nd
L (AD32)	9.10 ± 0.05	nd	nd	nd
M (AD22)	7.69 ± 0.23	nd	nd	nd
N (AD29)	4.78 ± 0.05	nd	nd	nd
O (AD32^*^)	14.60 ± 0.18	18.82 ± 0.03	1.083 ± 0.02	32.13 ± 0.01
(AD38)	9.50 ± 0.56	nd	nd	nd

*SOD, supeoxide dismutase; CAT, catalase; GSH, Gluatathione oxidase; nd, not detected. Values are the average of triplicates and were analyzed by Duncan Multiple Range Test at 5% real level*.

#### Antioxidant Enzymes

Four isolates, namely AD9, AD32, AD2, and AD26, produced varying amounts of antioxidant enzymes namely SOD, CAT, and GSH. The isolate AD32^*^ produced maximum activities of these SOD (18.82 ± 0.03 IU/mg), CAT (1.083 ± 0.02 mM of H_2_O_2_ decomposed/min), and GSH (32.13 ± 0.01 μM GSH reduced/min), respectively, compared to other isolates (Table 3).

### Plant Growth Promotion Studies

Among the various isolates, isolate AD32^*^ tolerated higher amounts of NaCl and produced copious amounts of plant growth-promoting traits and a good amount of salinity ameliorating metabolites, and hence it was used to evaluate its plant growth-promoting activities in rice. The effects of inoculation of these halophilic isolates on plant growth were visible on plant height, root length, plant dry weight, and number of tillers in rice seedlings at 21 DAS (Table 4). The inoculation of this halophilic PGPR in rice seedlings exhibited a significant (*p* < 0.05) improvement in the growth of rice plants under normal and salinity stress (8 d S/m -moderately saline) conditions. It enhanced 16% more seed germination (82%) under normal saline and 27% more seed germination over the control (60%) under salinity stress. It improved plant height by 11% under normal conditions and doubled the height under salinity stress. It resulted in an increase in 50% more root length, 25.00% more plant dry weight, and 41% more tillers in treated seedlings under salinity stress compared to the control and treated seedlings grown under normal soil. The inoculation of this halophilic isolated better plant growth under salinity stress.

**Table 4:** Plant growth promoting effects of halophillic isolates.

**Treatment**	**Seed germination**	**Plant height (cm)**	**Root length (cm)**	**Plant dry weight (mg)**	**Tiller (tiller/clump)**
Control	60%	2.87 ± 0.67^ab^	6.90 ± 0.46^a^	20.00 ± 2.00^abc^	19.00^a^
O (AD32^*^) under normal soil	71%	2.87 ± 0.67^ab^	6.90 ± 0.46^a^	20.00 ± 2.00^abc^	25.00^ab^
O(AD32^*^) under saline soil	87%	2.33 ± 0.21^a^	10.00 ± 0.70^abc^	19.67 ± 3.21^abc^	31.00^abc^

*Values are the mean of triplicates and were analyzed by Duncan Multiple Range Test at 5% real level. Figures followed by the same figures are not significantly different and figures followed by the different letters are statistically different*.

## Discussion

A wide range of PGPR, such as Streptomyces, Azospirillium, Clostridium, Alcaligenes, Bacillus, Pseudomonas, Thiobacillus, Serratia, Streptomyces strains, and so on, have been characterized for their role as PGPR during salt stress. Klebsiella is reported to promote plant growth under salt-stressed conditions by inducing physiological modifications in plants by varied mechanisms. Streptomyces strain was isolated by various scientists, which possesses various PGPR activities such as IAA production, P solubilization, and ACC deaminase activity (Salla et al., 2014; Passari et al., 2017; Etesami and Glick, 2020).

Passari et al. (2018) isolated 129 rhizospheric bacteria from *Curcuma longa*, and isolates exhibiting antagonistic potential were further screed for PGP traits. Two of the isolated strains identified as *Bacillus amyloliquefaciens* not only exhibited plant-promoting traits but also showed antimicrobial activity against fungal plant pathogens (Passari et al., 2018).

Palaniyandi et al. (2014) isolated 08 *Streptomyces* sp. strain and reported that A39 produces the highest amount of IAA (6.27 ± 0.42 μg/mL) and solubilizes a high amount of phosphate (91.79 ± 1.54 μg/mL) (Palaniyandi et al., 2014) even at higher salt concentration. Although phosphate solubilization was adversely affected at higher salt concentrations, *Streptomyces variabilis* (4NC), *S. fradiae* (8PK), and Streptomyces strain C-2012 have the potential for plant growth promotion and help plants to alleviate salt stress (Sadeghi et al., 2014; Vurukonda et al., 2018; Olanrewaju et al., 2019; Tolba et al., 2019). In the present study, AD29 tolerated 20% NaCl and showed higher IAA production compared to the other PGP traits. Phosphorus (P) is required to synthesize nucleic acid and energy-rich molecules as well as several biological processes. Phosphorus is present in an insoluble form, and plants cannot utilize it properly (Khan et al., 2020). In the present study, phosphate solubilization activity by the *Aspergillus terreus* clone ADCNM and *Aspergillus sp*. strain ADCNN were comparatively better at high salt concentrations. In a study, phosphate solubilization by *A. awamori* was recorded as 354.41 μg/mL (Saxena et al., 2016). Many microorganisms are studied to understand their phosphate solubilization capabilities, and *Bacillus* (Singh and Jha, 2016; Sarkar et al., 2018), *Enterobacter, Achromobacter xylosoxidans* (Ma et al., 2011), and *Delftia sp*. (Roy and Roy, 2019) can solubilize inorganic phosphate and synthesize organic acids such as the citric, gluconic, lactic, succinic, and propionic acids, which could be a mechanism to solubilize phosphate (Zheng et al., 2018). Acidification was the underlying mechanism for the solubilization of inorganic phosphate (Mendes et al., 2020).

Ammonia is better than nitrate or nitrite in the soil because ammonium has to be in a reduced state to get utilized by the plant. Ammonia is then converted to a utilizable form by the rhizoflora of the soil. In the present study, almost all isolates produced considerable ammonia when incubated on nitrogen-containing media, which suggests the PGP activities of the isolated microbes to acquire and provide nitrogen to the plant rhizospheres and increase the biomass (Marques et al., 2010; Chen et al., 2019).

Among the Gram-positive organisms, *Bacillus* sp. is the most dominant in all soil types, either in normal or saline-stressed conditions (Prittesh et al., 2020; Sultana et al., 2021). Salt tolerant microbes viz. *Bacillus aryabhattai, Achromobacter denitrificans*, and *Ochrobactrum intermedium* were isolated, which grew at 2.60 mol/L salt concentration and showed higher nitrogen fixation potential, phosphate solubilization, and IAA production at 200 mM salt stress (Sultana et al., 2021). Similarly, *Bacillus amyloliquefaciens* and *B. subtilis* were found to produce phytohormones such as gibberellins and indole acetic acid (Shao et al., 2015; Sorokan et al., 2021).

There were numerous attempts to identify and characterize salt-tolerant microbes from the rhizosphere, such as *Bacillus, Klebsiella, Agrobacterium, Pseudomonas, and Ochrobactrum sp*., and showed salt tolerance levels up to 10% NaCl (Upadhyay et al., 2012; Rajput et al., 2013; Sharma et al., 2015, 2016; Zheng et al., 2018; Chen et al., 2019). The current study results showed tolerance levels up to 20%. Moreover, these microbes also expressed their PGPR characteristic at elevated salt concentrations; hence, they have a high potential to be applied as biofertilizers. Amaresan and his coworkers had identified salt-tolerant (10% NaCl) plant growth-promoter organisms, viz. *Bacillus* spp.*, Alcaligenes faecalis, Enterobacter sp*. and *Lysinibacillus* sp. (Amaresan et al., 2016). All the isolated organisms showed varied levels of phosphate solubilization at 15% and 20% NaCl concentration. *Enterobacter sp* clone ADCNP and *Bacillus sonorensis* clone ADCN showed higher phosphate solubilization at higher salt stress. Nonetheless, in many isolates, the pH of the media and phosphate solubilization did not correlate (Tank and Saraf, 2010).

There are various mechanisms for phosphate solubilization, and one of the mechanisms is the production of organic acid and sugar utilization (Jeon et al., 2003). The isolated organisms grew on a wide pH range of 4 to 10; hence, at lower pH, some showed less response. Moreover, higher salt concentration with a prolonged incubation time is detrimental to the growth of the microbes. Therefore, *Bacillus* sp. clone ADCNA, *Bacillus megaterium, Bacillus* sp. clone ADCNE, and *Delftia* sp. clone ADCNK expressed lower phosphate solubilization at a higher salt concentration of 20%. PSBs increased the growth of the maize plant and its ability to acquire phosphorus when supplemented with phosphorus and varying concentrations of lime. The findings are suggestive of supplementing P along with the PSBs to improve plant yields and suggested that P should be applied from organic sources for the improvement of crop yield and P nutrition under saline/calcareous conditions (Adnan et al., 2020).

*A. ochraceus* produced 146 and 176 μg/mL IAA in 15% and 30% seawater (Badawy et al., 2021), respectively. However, in the present study, IAA production and siderophore production results were comparatively higher than the yield cited in previous studies at high salt concentrations. Siderophore production is one of the important plant growth-promoting traits, and AD2 produced 16.02% siderophore. The importance of siderophore production by *Bacillus subtilis* LSBS2 was explained through a pot culture experiment in *Sesamum indicum* L, leading to an improvement in growth parameters and nutrient content of plant and soil. A growth promoter strain *Aspergillus terreus* JF27 was isolated, which produced 17.5 μg/mL of IAA but could not solubilize phosphate in normal soil (Nithyapriya et al., 2021). *Aspergillus terreus* clone ADCNM and *Aspergillus* sp. strain ADCNN isolated in the current experiment showed higher salt tolerance up to 20%. *A. aculeatus* was inoculated with perennial ryegrass at different salinity levels. In this experiment, *Aspergillus* sp. assisted plant growth and alleviated stress as it tends to solubilize phosphorus and accelerate its uptake and utilization during stress conditions (Li et al., 2021). In some previous reports, researchers isolated and characterized 12 morphologically distinct *Aspergillus sp*. and obtained IAA production (6–96 μg/mL), GA (48–184.11 μg/mL), and siderophore (32–87% SU) production by the isolates (Pandya et al., 2018). Siderophore-producing *Aspergillus sp*. represses pathogen growth by utilizing iron available in the soil (Shaikh et al., 2014). ACC deaminase enzymatic activity exhibited by PGPR plays a key role in stimulating plant growth and withstanding stress conditions (Chandra et al., 2018). ACC secreted during stressed conditions is broken down by the enzyme ACC deaminase into ammonia and α-ketobutyrate, causing a major impact on plants physiology, growth, and development (Barnawal et al., 2014; Saleem et al., 2018). In a study, *Streptomyces* sp. GMKU 336 combated salt tolerance and enhanced growth in rice was supposedly due to its ability to produce ACC deaminase and consequently reduced ethylene concentration, exhibiting ROS activity (Jaemsaeng et al., 2018). In another such study, inoculation of bacterial isolates *Aneurinibacillus aneurinilyticus* and *Paenibacillus* sp., in consortia, increased the root length and shoot length their weight and biomass in *Phaseolus vulgaris* seedlings in saline-stressed conditions (Gupta and Pandey, 2019).

There are many pathways, directly and indirectly, exhibited by PGPR in improving crop yields in salt-stressed conditions, including the production of ACCD (Sagar et al., 2020). The enzyme ACCD lowers the level of ACC in root exudates; the suboptimal level of ACC reduces the concentration of ethylene in the plant roots and thus helps in root length, which improves the absorption of nutrients (Kusale et al., 2021a,b; Sagar et al., 2022a,b). Many ACCD-producing PGPR are known to ameliorate salinity stress in plants (Kusale et al., 2021a). These isolates grew well at high salt levels, exhibited optimum ACCD activity at high salt levels (15% NaCl), and helped ameliorate salt stress in rice seedlings.

Excess salt conditions impose oxidative stress that damages the plant cell membranes and cell structures resulting in decreased growth and making the plants sensitive to pathogen attack. Antioxidant enzymes such as SOD, CAT, and GSH producing PGPR (Acuña et al., 2019) protect plants from oxidation due to osmotic shocks caused by excess salts (Fazeli and Sayyed, 2019). Under excess salt conditions, these PGPR activate an antioxidative defensive system in the crops and helps remove the free radicals produced due to salt (Acuña et al., 2019). Sapre et al. (2018) reported halophilic *Klebsiella* sp. that tolerated high salt concentration and produced antioxidant enzymes under salt stress conditions.

The overall growth, development, and health of crop plants depend on the availability of sufficient amounts of nutrients and it is regulated by the ability of its microbiome to produce a variety of plant growth-promoting traits to improve plant growth (Khumairah et al., 2018). Inoculation of multifarious rhizobacteria provides multiple nutrients to the plants and thus helps grow plants (Gupta and Pandey, 2019; Alishahi et al., 2020).

Most of the saline soils are deficient in nutrient content. Consequently, an integrated crop and soil nutrient management through the application of multifarious halophillic PGPR with salinity ameliorant potential can help in managing the biodiversity of microbial fertilizers, improve soil nutrients, alleviate salinity stress, and sustainable crop growth under salt stress. These studies indicate the role of various mechanisms at the molecular level during the plant-PGPR interactions. Further study of the mechanisms and experimenting with these organisms in different plants would lead to the efficient use of these PGPRs as bioinoculants for soil salinity mitigation. Many such investigations and experiments conclude that PGPB is a potential alternative to withstand abiotic stress in plants and enhance plant growth promoters.

## Conclusion

Salinization of soil influences plant growth and development in terms of quantitative production as well as the quality of the growth. Plants are many times adaptive to withstand these salt stressors due to the metabolic activities of the plant growth-promoting microorganisms, the interplay of biomolecules, and the biochemical changes which result in combating these adverse conditions. Due to its multiple PGP traits, these halo tolerant microbial populations have diversified physiological and biochemical adaptations while residing in the rhizosphere. The diversified mechanisms of these PGP microbes boost plant growth and development even in salt stress conditions. The current study has demonstrated the positive effect of salinity on plant growth and development by combinations of PGP traits such as increased phosphate solubilization, organic acid modulation of phytohormones, siderophore production, secretion of organic acids, and biofilm formation enabling to combat the salt stress. Hence, a consortium of the PGPs and its interactive effect needs to be studied at the molecular level to understand the existence of microbial communities in different conditions and its effect in field conditions.

## Data Availability Statement

The original contributions presented in the study are included in the article/supplementary material, further inquiries can be directed to the corresponding authors.

## Author Contributions

NP: conceptualization and supervision. AR: methodology. CK and HV: formal analysis. NP, AR, and RZS: writing—original draft. RZS, HV, MJA, AG, and PP: review and editing. SA and PP: funding acquisition. All authors have read and agreed to the published version of the manuscript.

## Funding

This project was supported by Researchers Supporting Project Number (RSP-2023R7) King Saud University, Riyadh, Saudi Arabia. Open access funding by the University of Helsinki, Helsinki, Finland.

## Conflict of Interest

The authors declare that the research was conducted in the absence of any commercial or financial relationships that could be construed as a potential conflict of interest.

## Publisher's Note

All claims expressed in this article are solely those of the authors and do not necessarily represent those of their affiliated organizations, or those of the publisher, the editors and the reviewers. Any product that may be evaluated in this article, or claim that may be made by its manufacturer, is not guaranteed or endorsed by the publisher.
